# Supplementary material for: Insights into the self-assembly and interaction of sars-cov-2 fusion peptides with biomimetic plasma membranes
Source: Nat Commun. 2025 Dec 20;17:915. doi: 10.1038/s41467-025-67640-5 (PMC12830992; doi:10.1038/s41467-025-67640-5)
Supplement: Supplementary file 1 — Supplementary Information [file 41467_2025_67640_MOESM1_ESM.pdf]

-Supporting Information-

Insights into the Self-Assembly and Interaction of SARS-CoV-2 Fusion Peptides with Biomimetic Plasma Membranes

Nisha Pawar,[1] Andreas Santamaria,[2,3] Brigida Romano,[2] Krishna C. Batchu,[2] Valerie Laux,[2] Eduardo Guzman,[3,4] Nathan. R. Zaccai,[5] Alberto Alvarez-Fernandez,[1,*] Armando Maestro.[1,6,*]

[1] *Centro de Física de Materiales (CFM) (CSIC–UPV/EHU) – Materials Physics Center (MPC), Paseo Manuel de Lardizabal 5, San Sebastián, 20018 Spain*

[2] *Institut Laue-Langevin, 71 Avenue des Martyrs, 38042 Grenoble, Cedex 9, France.*

[3] *Departamento de Química Física, Facultad de Ciencias Químicas, Universidad Complutense de Madrid, Ciudad Universitaria s/n, 28040, Madrid, Spain*

[4] *Instituto Pluridisciplinar, Universidad Complutense de Madrid, Paseo Juan XXIII 1, 28040, Madrid, Spain*

[5] *Cambridge Institute for Medical Research, University of Cambridge, Cambridge CB22 7QQ, United Kingdom.*

[6] *IKERBASQUE-Basque Foundation for Science, Plaza Euskadi 5, Bilbao, 48009, Spain*

*E-mail address: alberto.alvarez@ehu.eus; armando.maestro@ehu.eus

Content.

Supplementary Figures……………………………………………..Page 2

Supplementary Tables………………………………………………Page 10

Supplementary Methods……………………………………………Page 15

Supplementary References…………………………………………Page 18

1. Supplementary figures.

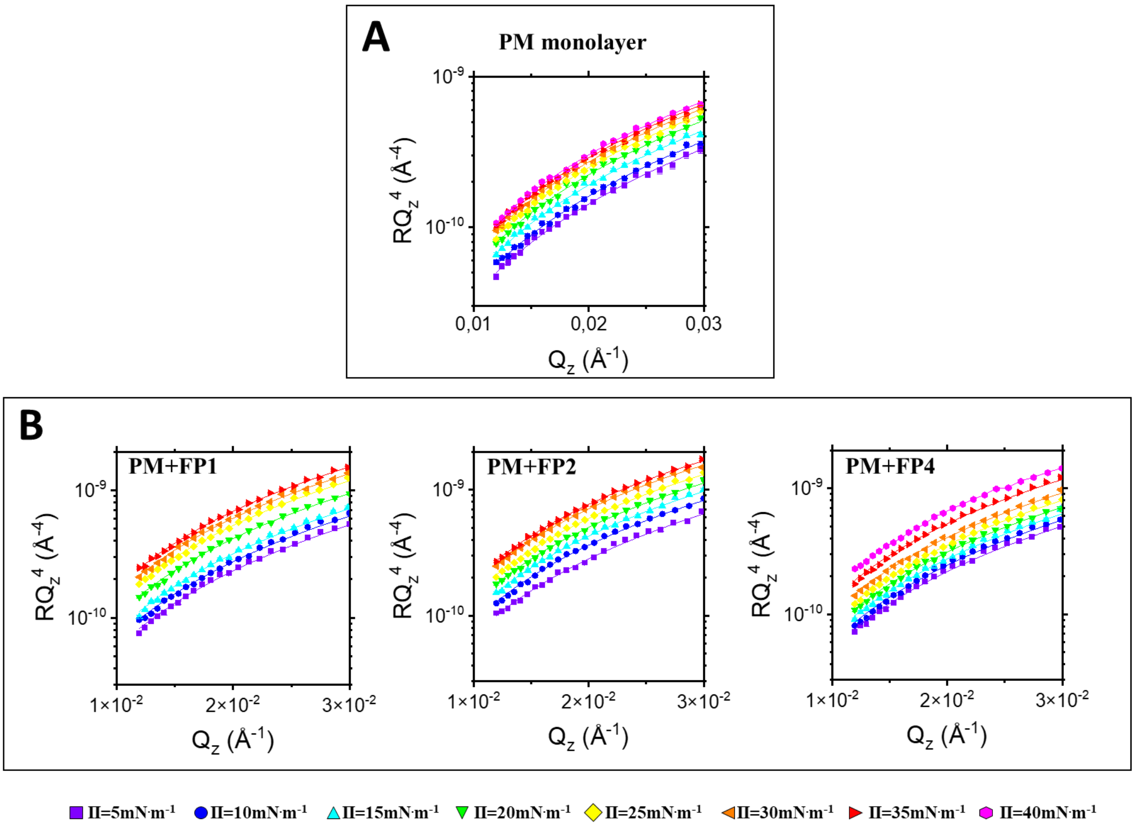

Supplementary Figure 1. NR data acquired at a restricted low-Q_z at different surface pressures and the corresponding fitting curves (lines): **A** PM, **B** PM-FP1, PM-FP2, and PM-FP4 monolayers. The data were recorded in ACMW (8% D_2O, SLD = 0).

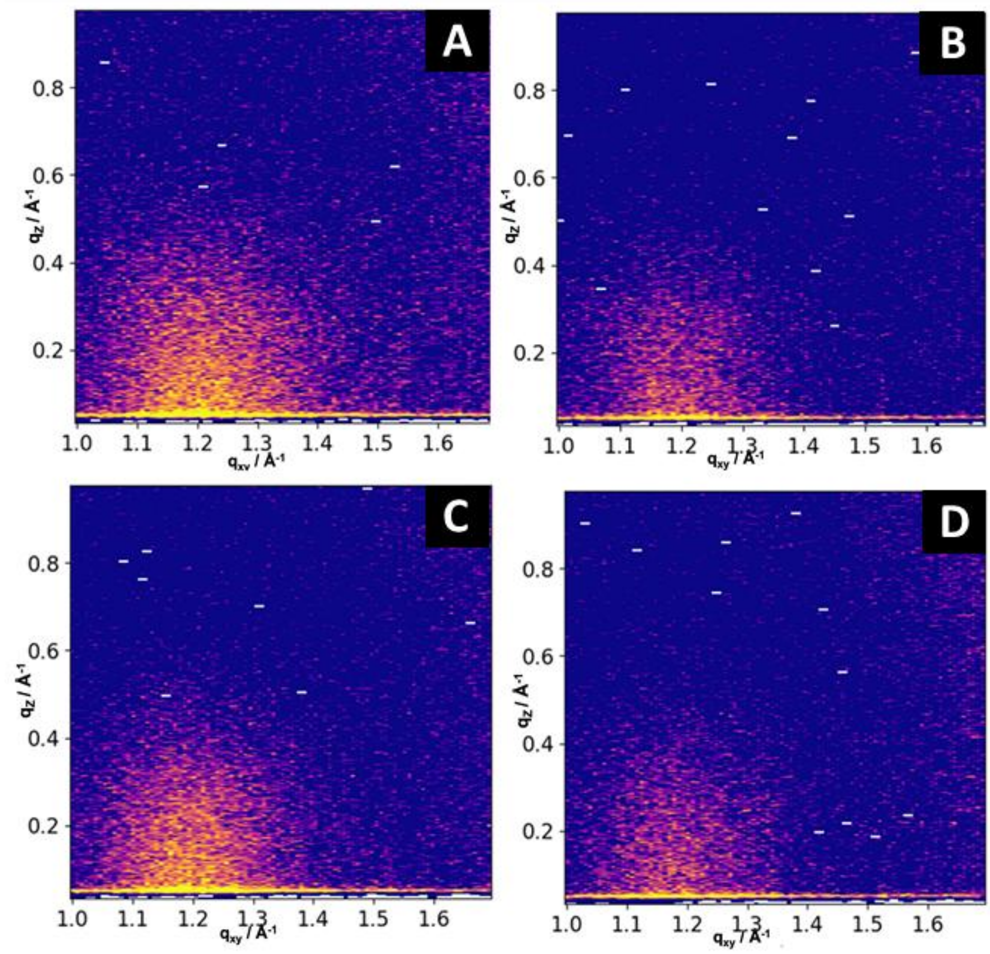

Supplementary Figure 2. GIXD contour plot for the PM monolayer (A), PM-FP1 (B), PM-FP2 (C), and PM-FP4 (D) at a surface pressure of 20 mN/m.

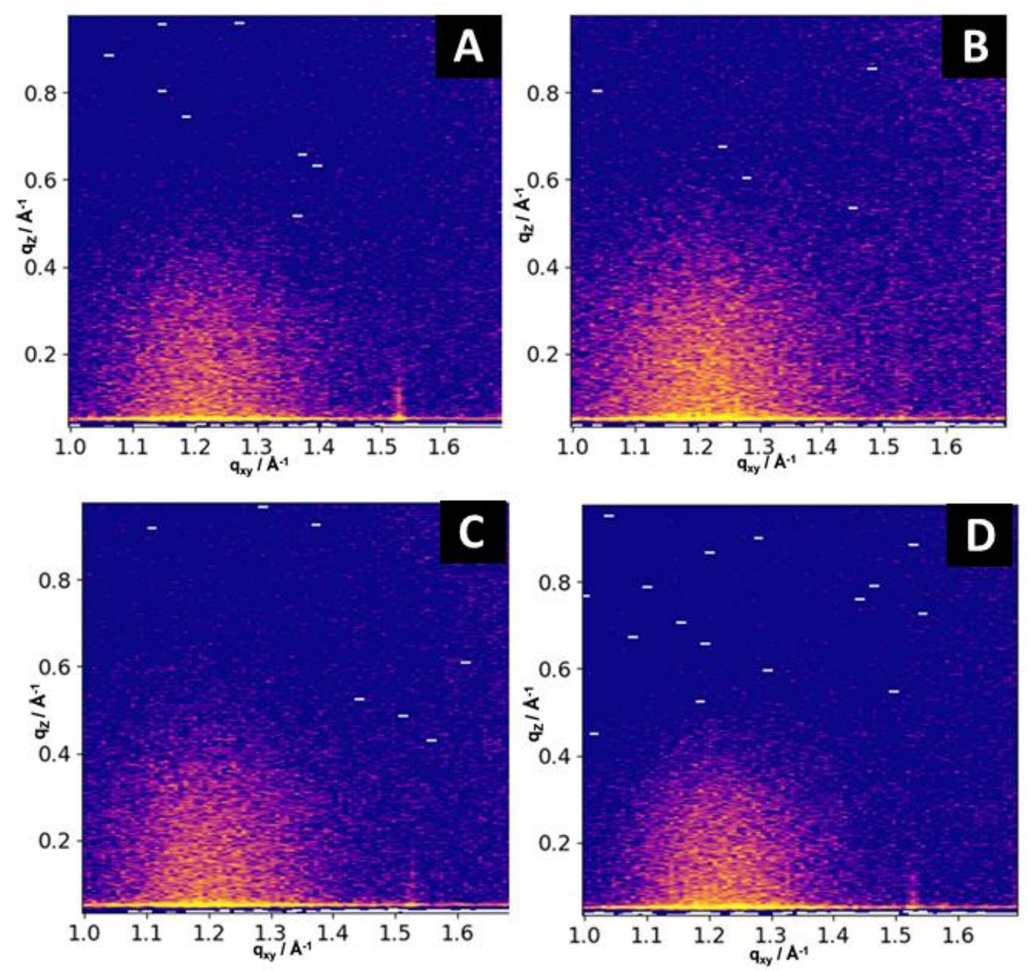

Supplementary Figure 3. GIXD contour plot for the PM monolayer (A), PM-FP1 (B), PM-FP2 (C), and PM-FP4 (D) at a surface pressure of 35 mN/m

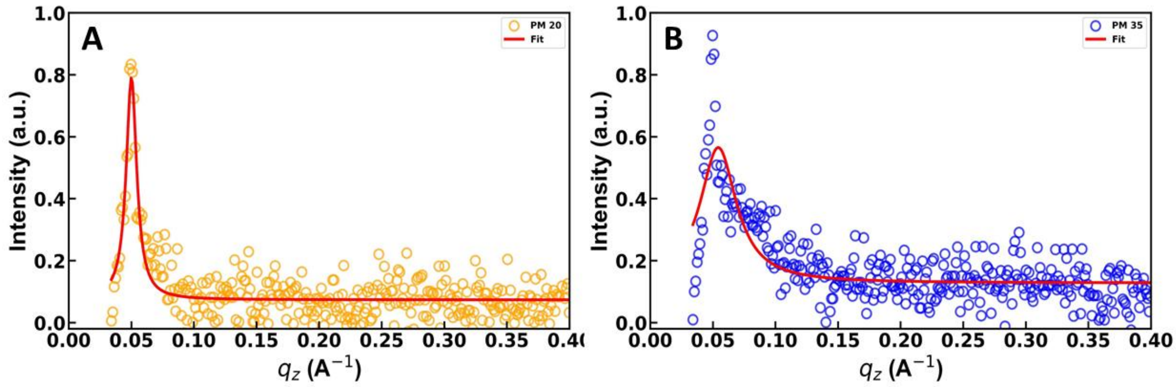

Supplementary Figure 4. Variation of diffracted intensity as a function of out of -plane scattering vector component (q_z) from PM at 20 mN/m (A) and 35mN/m (B).

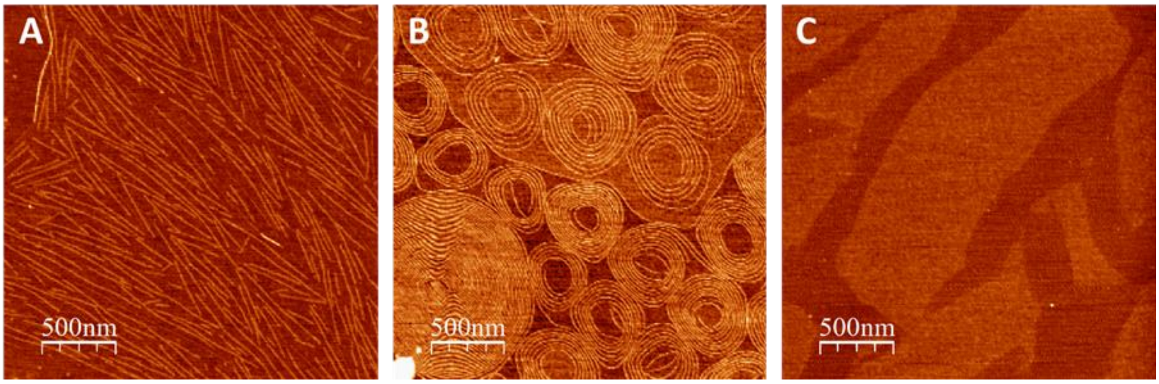

Supplementary Figure 5. AFM topographical micrographs of PM-FP1 (A), PM-FP2 (B), and PM-FP4 (C) at 20 mN/m surface pressure onto mica substrate.

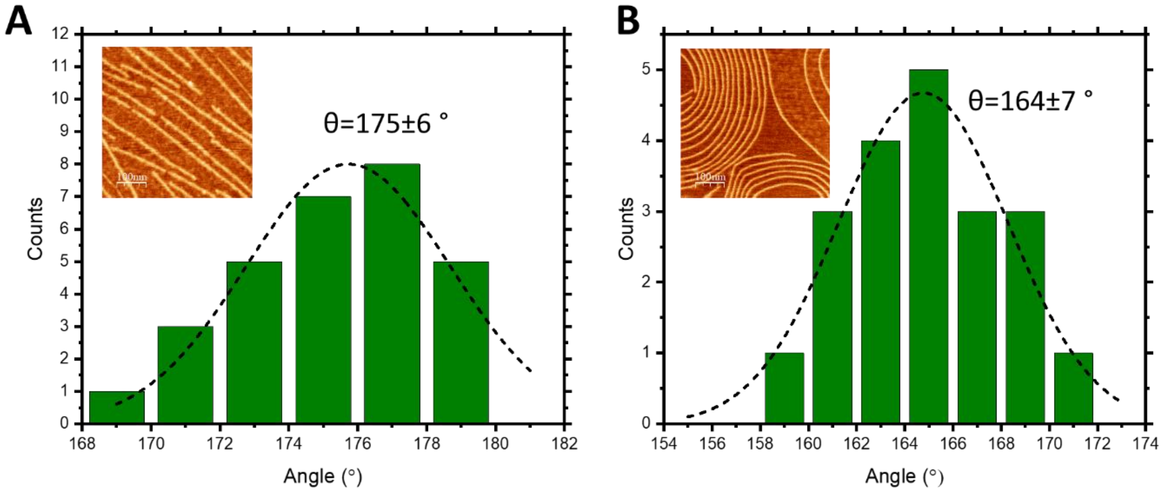

Supplementary Figure 6. Fibres curvature angle distributions for PM-FP1 (A) and PM-FP2 (B) samples compressed at 20 mN/m

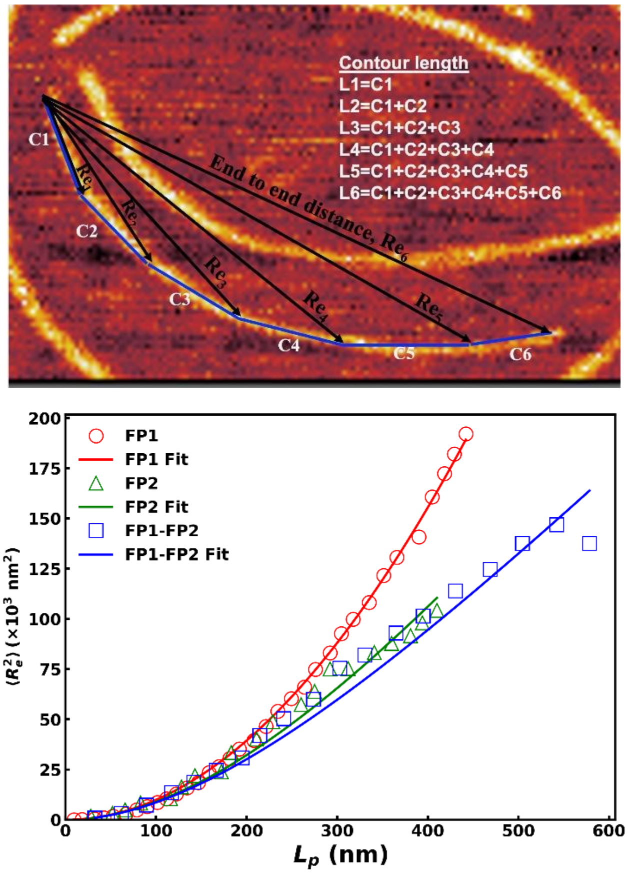

Supplementary Figure 7. (Top) Cartoon to show the determination of end-to-end distance and contour length. (Bottom) Variation of the mean squared end-to-end distance of peptide fiber (FP1, FP2 and tandem FP1-2) on the PM as a function of their contour length. The solid lines represent the fitting of data with Equation S1.

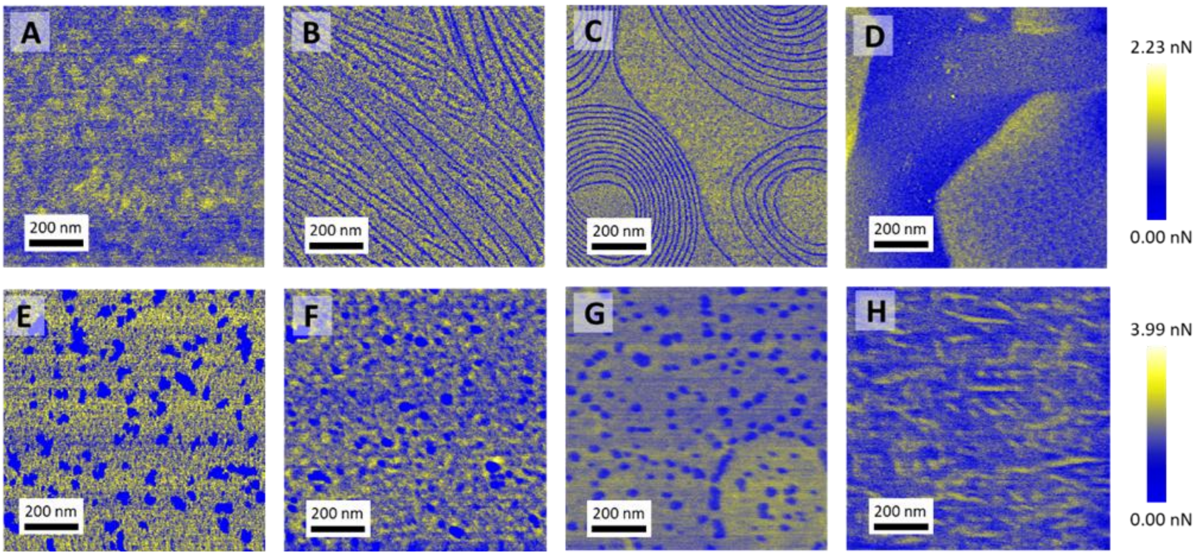

Supplementary Figure 8. AFM adhesive force maps of the pristine PM monolayer (A, E), PM-FP1 (B, F), PM-FP2 (C, G), and PM-FP4 (D, H) at 20 mN/m (top) and 35 mN/m (bottom) surface pressure onto mica substrate.

2. Supplementary Tables.

Supplementary Table 1. Persistence length results obtained from the AFM micrographs.

	I x 10^{-36} (m^4)	Persistence length (L_p) nm	Young Modulus (Y) (GPa)
PM - FP1	1.14 ± 0.03	2320 ± 290	8.3 ± 0.4
PM - FP2	7.80 ± 0.02	145 ± 9	0.80 ± 0.01
PM – FP1-2	3.97 ± 0.02	109 ± 6	0.11± 0.01

Supplementary Table 2. Physicochemical properties of the peptide investigated.

Peptide	Sequence	Number of amino acids	MW (Da)	Net charge
**FP1**	816-SFIEDLLFNKVTLADAGFIKQY-837	22	2538	-1
**FP2**	835-KQYGDCLGDIAARDLICAQKFN-856	22	2456	0
**FP4**	885-GWTFGAGAALQIPFAMQMAYRFNGI-909	25	2723	+1

Supplementary Table 3. Biomimetic plasma membrane composition used in this study.

**Composition in mol%**	**Extracted Natural Lipids**					
	PC pool	*PE pool*	*PS pool*	*PI pool*	*Sphingomyelin*	*Cholesterol*
**PM**	20	11	6	0	13	50

**Phospholipid acyl chain composition in mol%**	**Hydrogenous**		**Deuterated**	
	**16C**	**18C**	**16C**	**18C**
**PM**	35.3	62.6	29.6	68.3

Supplementary Table 4. Fixed parameters used in the NR data analysis: SLD, scattering length values (b), interfacial roughness (r) and volume fraction of water in the monolayer (f_w).

Fixed Parameters	
$b_{d\text{-}PM}$ (10^{-5} Å)	528.46
$SLD_{d\text{-}PM}$ (10^{-6} Å^{-2})	5
b_{FP1} (10^{-5} Å)	590.5
b_{FP2} (10^{-5} Å)	582.1
b_{FP4} (10^{-5} Å)	669.0
r (Å)	3
f_w (0:1)	0

Supplementary Table 5. Low-q_z NR data analysis: Scattering length density (ρ) times thickness of the monolayer (t) composed of PM and FPs.

**Π (mN·m^{-1})**	**ρt (10^{-6} Å^{-1})**			
	PM / ±0.3	PM-FP1 / ±0.3	PM-FP2 / ±0.3	PM-FP4 / ±0.3
5	77.3	60.1	66.3	58.2
10	82.9	65.5	75.4	61.7
15	88.7	70.3	82.2	65.4
20	96.3	81.0	96.7	69.7
25	101.2	91.3	105.7	74.4
30	105.4	97.6	110.3	80.0
35	108.8	104.0	133.2	90.9
40	11.5	127.8	//	121.4

3. Supplementary Methods.

Persistence length calculation

We modelled FP1 and FP2 peptide fibrils observed at the plasma membrane (PM) surface as worm-like chains, following the worm-like chain model of flexible polymers. Using this model, we measured the persistence length of the fibrils based on their shape, specifically by analyzing the relationship between their contour length (L), and end-to-end distance.[1] Thus, FP fiber was divided into "n" number of individual nanorods (for our analysis n=30).

Supplementary Figure 7A illustrates the schematic representation used to determine the end-to-end distance (Re) as a function of the fiber's contour length (Lc). In this schematic, the fiber is divided into six individual segments (C1–C6), each with a specific contour length. The corresponding end-to-end distances (L1–L6) for different contour lengths were measured using Gwyddion software. Assuming that the individual nanorods conform to the worm-like chain (WLC) model, the relationship between Re and Lc for a peptide fiber was analyzed as described in references.[1–3] The persistence length (PPP), as defined by **Equation S1**,[1–3] was calculated from AFM images, assuming that peptide fibers behave as nanorods with a circular cross-section. **Supplementary Figure 7B** displays the variation of the mean squared end-to-end distance of peptide fibers (FP1 and FP2) on the PM as a function of contour length. The data in Figure S6B were fitted to **Equation S1**, yielding the persistence length of the fibers. The calculated persistence length values for FP1 and FP2 at the monolayer of PM are presented in **Supplementary Table 1**.

$$\langle R_e^2 \rangle = 4L_p\left[L_c - 2L_p\left(1 - e^{-L_c/2L_p}\right)\right] \qquad \textbf{Equation S1}$$

Using the average AFM height (d), we determine the moment of inertia ($I=\pi d^4/64$). This was subsequently used to determine the Young modulus (Y) of the peptide as given by **Equation S2**.

$$\mathrm{Y} = \frac{\text{bending stiffness}}{\mathrm{I}} = \frac{L_p \mathrm{k_B T}}{\mathrm{I}} \qquad \textbf{Equation S2}$$

The results presented in **Table S1** indicate that FP2 has a lower persistence length compared to FP1, which corresponds to its greater flexibility and ability to form spirals on the plasma membrane (PM). The values of Young modulus obtained from FP1 and FP2 are in well confirmation with the measured value DMT modulus from AFM Peak force measurements.

Bending Rigidity and Spiral Energy of FP2 and FP1-2 Fibers

This note provides a detailed step-by-step calculation of the mechanical properties of FP2 and the tandem FP1–2 fusion peptide fibers bending rigidity, and spiral bending energy from the parameters obtained from the AFM analysis reported in **Table S1**. These properties help understand how FP2 and FP1-FP2 tandem contributes to membrane curvature and viral fusion mechanisms.

Bending rigidity (κ) from Elastic Beam theory:

$$\kappa = \frac{YI}{(1-\nu^2)}$$ **Equation S3**

where ν is the Poisson ratio. In the calculation of bending rigidity, we assume a Poisson's ratio of 0.5, corresponding to the incompressible limit. This is appropriate for hydrated, soft peptide assemblies such as the β-sheet-rich FP2 fibers observed here, which deform primarily through bending with negligible volume change. Similar assumptions have been adopted in mechanical analyses of amyloid fibrils and other supramolecular biomaterials.[4]

$$\kappa_{FP2} \approx 0.8 \times 10^9 \times 7.80 \times 10^{-36} / 0.75 \approx 8.32 \times 10^{-27}\ \mathrm{Nm^2}$$

$$\kappa_{FP1} \approx 8.3 \times 10^9 \times 1.14 \times 10^{-36} / 0.75 \approx 12.6 \times 10^{-27}\ \mathrm{Nm^2}$$

$$\kappa_{FP1-2} \approx 0.11 \times 10^9 \times 3.97 \times 10^{-36} / 0.75 \approx 5.93 \times 10^{-28}\mathrm{Nm^2}$$

Spiral Geometry and Bending Energy of FP2

FP2 fibers form well-defined Archimedean spiral structures with the following geometric parameters obtained from AFM:

- Inner radius (r_{in} = 100 nm),

- Outer radius(r_{out} = 400 nm)

- Pitch (P = 30 nm)

  - Spiral growth rate: a ≈ 4.77 nm/rad
  - Number of turns: N ≈ 10

Determination of Arc Length:

$$\theta_{max} = \frac{r_{out} - r_{in}}{a} \approx 62.9 \text{ rad} \qquad \textbf{Equation S4}$$

To estimate the total length of an FP2 spiral fiber, we model its geometry as an *Archimedean spiral*, where the radius increases linearly with the angular coordinate according to the equation:

$$r(\theta) = r_{in} + a\theta \qquad \textbf{Equation S5}$$

The total arc length **S** of the spiral is calculated using the following standard expression for Archimedean spirals [Weisstein, E. W. *"Archimedean Spiral."* From MathWorld A Wolfram Web Resource. https://mathworld.wolfram.com/ArchimedeanSpiral.html]:

$$S = \int_0^{\theta_{max}} \sqrt{r(\theta)^2 + \left(\frac{dr}{d\theta}\right)^2}\, d\theta = \int_0^{\theta_{max}} \sqrt{(r_{in} + a\theta)^2 + a^2 d\theta} \qquad \textbf{Equation S6}$$

This integral was evaluated numerically, giving a total spiral length of approximately S= 7.25 µm, which is in line with the observed fibrillar features in AFM topographic images.

<u>Spiral Bending Energy Calculation</u>

The local curvature of the spiral varies along its arc length. To compute the total bending energy, we integrate the curvature-dependent energy density along the spiral contour:

$$B = \frac{1}{2}\kappa \int_0^S \left(\frac{1}{R(s)}\right)^2 ds \qquad \textbf{Equation S7}$$

Here, R(s) is the local radius of curvature as a function of arc length s, and S is the total spiral length. This integral is evaluated numerically using the geometric parameters derived above.

We use the values of κ, obtained from the elastic beam theory expression

$$B_{FP2} \approx 1.05 \times 10^{-19} \text{ J}$$

$$B_{FP2}/_{KT} \approx 25.5 \text{ KT}$$

Assuming same spiral geometry as FP2:

$$B_{FP1-2} \approx 0.75 \times 10^{-19} \text{ J}$$

$$B_{FP1-2}/_{KT} \approx 18.3 \text{ KT}$$

4. Supplementary References

[1] C. Rivetti, M. Guthold, C. Bustamante, “Scanning Force Microscopy of DNA Deposited onto Mica: EquilibrationversusKinetic Trapping Studied by Statistical Polymer Chain Analysis” *J Mol Biol* **1996**, *264*, 919–932.

[2] D. Murugesapillai, S. Bouaziz, L. J. Maher, N. E. Israeloff, C. E. Cameron, M. C. Williams, “Accurate nanoscale flexibility measurement of DNA and DNA–protein complexes by atomic force microscopy in liquid” *Nanoscale* **2017**, *9*, 11327–11337.

[3] F. Gittes, B. Mickey, J. Nettleton, J. Howard, “Flexural rigidity of microtubules and actin filaments measured from thermal fluctuations in shape.” *J Cell Biol* **1993**, *120*, 923–934.

[4] T. P. Knowles, A. W. Fitzpatrick, S. Meehan, H. R. Mott, M. Vendruscolo, C. M. Dobson, M. E. Welland, “Role of Intermolecular Forces in Defining Material Properties of Protein Nanofibrils” *Science (1979)* **2007**, *318*, 1900–1903.
